# Scaffolding layered control architectures through constraint closure: insights into brain evolution and development

**DOI:** 10.1098/rstb.2020.0519

**Published:** 2022-02-14

**Authors:** Stuart P. Wilson, Tony J. Prescott

**Affiliations:** ^1^ Department of Psychology, University of Sheffield, Sheffield, UK; ^2^ Department of Computer Science, University of Sheffield, Sheffield, UK

**Keywords:** constraint closure, brain evo-devo, hierarchical control, Baldwin effect, social thermoregulation, visual attention

## Abstract

The functional organization of the mammalian brain can be considered to form a layered control architecture, but how this complex system has emerged through evolution and is constructed during development remains a puzzle. Here we consider brain organization through the framework of constraint closure, viewed as a general characteristic of living systems, that they are composed of multiple sub-systems that constrain each other at different timescales. We do so by developing a new formalism for constraint closure, inspired by a previous model showing how within-lifetime dynamics can constrain between-lifetime dynamics, and we demonstrate how this interaction can be generalized to multi-layered systems. Through this model, we consider brain organization in the context of two major examples of constraint closure—physiological regulation and visual orienting. Our analysis draws attention to the capacity of layered brain architectures to scaffold themselves across multiple timescales, including the ability of cortical processes to constrain the evolution of sub-cortical processes, and of the latter to constrain the space in which cortical systems self-organize and refine themselves.

This article is part of the theme issue ‘Systems neuroscience through the lens of evolutionary theory’.

## 1. Introduction

Beginning with the nineteenth century philosopher, Herbert Spencer [1], and the neurologist, John Hughlings Jackson [2,3], there is an extensive literature that views the nervous system as a layered architecture (for reviews, see [4–10]). The notion of layering implies a vertical decomposition of control similar to the concept of hierarchical organization, but without an insistence on a unidirectional (top-down) flow of control. As stressed by Jackson, the concept of layering also emphasizes the capacity of lower level systems to operate in the absence of higher levels, either during development, or following brain damage [4]. Evolutionary neurobiology confirms an increase in layering with increased brain size [11], a trend which is recapitulated developmentally [12–14], with disproportionate increase in the size of late-developing structures [15]. Notably, in systems engineering, layered architectures are seen as an effective means to implement robust and accurate control using components that may be individually slow or noisy [16–21].

In human evolution, the trend towards increased brain size has manifested through an increased size of the cortex, particularly frontal, parietal and temporal areas [11,22], and a corresponding extended period of postnatal development of these brain regions [22], during which interactions with the child’s physical and social world help to shape and refine neural processing. Johnson [23–26] summarizes postnatal brain development in humans as a process of ‘interactive specialization’ that involves increased specialization of cortical areas and fine-tuning of responses. According to this view, cortical development is partly a competitive process in which initial regional biases become amplified as local networks adapt to specialize for specific computational roles. This adaptation is both activity-dependent and relies on interactions both within cortex and with subcortical substrates. In this contribution, we will explore some of the implications of this view, using the specific example of visual orienting.

More broadly, the capacity to cope adaptively with changing environments is critical to living systems and to their need to maintain order in the face of the thermodynamic tendency towards increased entropy. Robustness to adverse events is critical to survival, and, as with the design of many modern engineered systems [19], biological controllers have evolved to be risk-sensitive (including to worst-case scenarios) rather than simply optimal for the average setting [27]. As we will see, robustness is provided by multiple parallel regulatory pathways with some capacity to operate independently, thus showing evidence of a layered architecture.

A key notion in biological regulation is Cannon’s idea of homeostasis [28], as the maintenance of a stable internal state in the context of perturbation. Though often characterized in terms of feedback control, homeostasis includes the idea of predictive control, that is, acting in advance of an anticipated perturbation to reduce its effects [29]. The concept of allostasis, originated by Sterling [30,31], extends this notion of adaptive regulation by recognizing that the internal balance may shift with circumstances, for example, throughout the day, or with the organism’s bodily or environmental context. Both homeostasis and allostasis emphasize the maintenance of equilibria, and while this has sometimes been expressed through the engineering idea of ‘set points’, the search for brain mechanisms that explicitly defend specific values or ranges of key physiological variables has yielded little evidence [29,32], leading to the alternative idea of ‘balance points’ as values that are implicitly maintained through the interacting dynamics of multiple regulatory processes [29,33]. Further, allostatic systems are sometimes thought of as guiding a to-be-regulated homeostatic system by modifying its target state, presumably to minimize some discrepancy signal defined at the level of the allostatic controller [30]. Again, this idea may need to be reconsidered given the more holistic view of physiological regulation that is emerging in the literature [29,34].

In both the interactive specialization view of cortical development, and in the emerging consensus on the distributed nature of physiological regulation, we see a broader principle at work—that components of biological systems maintain a circular dependency referred to as *constraint closure*. In this contribution, we will explore this idea in more detail and with respect to its implications for understanding the evolution of the brain as a layered control architecture.

## 2. Constraint closure

Constraint closure describes a general theory of biological organization, which has recently been gaining traction [35–40]. Building on the related concept of autopoeisis [41], the key idea is that biological systems are composed of interacting processes and can maintain their dynamics if those processes are mutually constraining. Constraint closure additionally requires that the mutually constraining processes have dynamics that fundamentally operate on different timescales.

From this perspective, Montévil & Mossio [35] outline the necessary conditions for biological organization as follows. First, a *constraint* is something that (i) modifies a process on the characteristic timescale of the dynamics of that process, but (ii) is not itself modified at the timescale of that process. As such, an enzyme can be a constraint for a chemical reaction, to the extent that it catalyses that reaction without being fundamentally altered by it. Second, if X is a constraint on process *A*, and Y is a constraint on process *B*, and if Y is also a product of process *A*, then constraint Y is said to be *dependent* on constraint X, and constraint X is said to be *generative* for constraint Y. Third, constraint closure is realized when, for each in a set of constraints, the constraint is both (strictly) dependent on another constraint (the relevant timescale of its dynamics is slower) and generative for another (the relative timescale of its dynamics is faster). A key proposal is that a system which is organized in this way will be capable of maintaining dynamic stability.

The process of operationalizing a theory, by specifying its assumptions explicitly as terms in a computational model, can help provide new ways of testing those assumptions, and can often illuminate unforeseen consequences of those assumptions. As we will see in the following sections, operationalizing the concept of constraint closure through a computational model reveals how the dynamics of faster processes can serve to *scaffold* the dynamics of slower processes, permitting systems of mutually interacting slow and fast processes to discover useful states that may otherwise be impossible for a system to discover. Drawing on the insights developed through this model, we will then explore how neural and behavioural processes may serve as scaffolds for one another in the evolution and development of the brain.

## 3. Towards a computational model of constraint closure

The key assumption of constraint closure is that processes with dynamics operating on different timescales mutually constrain each other. For fast processes to constrain slow processes, and thus for constraint closure to be achieved, some accumulated product of a fast process must, at least implicitly, be measured by the slow process; some signal must be integrated over time. An account of how the necessary temporal integration can occur, or why we might expect the constraint of slow processes by fast processes to be pervasive in natural systems, has yet to be fully developed.

### (a) How learning can constrain evolution

Thinking about this particular problem, and more specifically thinking about how a fast process may *usefully* constrain a slow process, led us back to a seminal paper by Hinton & Nowlan [42], in which mutually constraining dynamical interactions between processes operating on different (phylogenetic and ontogenetic) timescales were modelled explicitly. In their paper, which is considered by many to capture the essence of the Baldwin effect [43,44], a computer model was used to operationalize the idea that learning, which occurs within a lifetime, can accelerate evolution by natural selection, which occurs between lifetimes, without information about what has been learned being passed on directly from one lifetime to the next. If we consider learning to be a fast process, and natural selection to be a comparatively slow process, then this operationalized description of the Baldwin effect may be useful for understanding how fast processes can constrain slower processes in more general terms. So here we briefly outline the original model before exploring how a generalization of this scheme can promote our understanding of constraint closure in biological systems.

The idea of the Baldwin effect is that once the ability to learn a ‘good trick’ [45] emerges in a population, a new selection pressure to learn the trick more efficiently arises. Natural selection will thereafter favour genetic variations that increasingly consolidate the trick (facilitate its acquisition), to the extent that, over generations, performing the trick becomes an essentially innate ability. Hinton & Nowlan’s model demonstrates that the Baldwin effect requires the process of acquiring the trick, i.e. learning, to be costly, such that genetic consolidation of the trick occurs via selection pressure on the costs associated with prolonged or inefficient acquisition.

They defined a population of 20-character strings, where characters ‘1’ and ‘0’ correspond to genetically fixed alleles, and the character ‘?’ can temporarily adopt random binary states in 1000 iterations representing lifetime learning. They defined a state of all ‘1’ characters to be maximally fit, and all other states to be equally unfit. In the non-learning case, where there are no ‘?’ characters in the population, the chance of being in the target state is 1/2^20^ and using a genetic algorithm (recombination without mutation in a population of size 1000) to search for it in this space is nearly impossible, like trying to find a needle in the proverbial haystack. But in the learning case, where a proportion of the characters in the initial population (say 50%) are ‘?’, evolution becomes possible—within 100 generations or so, the population converges to a state in which all individuals are composed of ‘1’ or ‘?’ characters only, and within each lifetime (of random learning trials) the fit state is consistently acquired.

Hinton & Nowlan explain that learning, here the capacity to flip the state of the ‘?’ characters, smooths the fitness landscape and creates a ‘zone of increased fitness’ around the target state, which is graded and so makes the search for peak fitness via gradient ascent tractable. Crucially, the binary states that the ‘?’ characters adopt during the lifetime of learning are not passed on directly from one generation to the next, representing an assumption that information may only flow from genotype to phenotype. Rather, individuals that have the *opportunity* to acquire the target state, i.e. those comprising characters ‘1’ or ‘?’ only, are favoured in the selection process, and those opportunist individuals that acquire the target state more rapidly are increasingly favoured. Once an individual in the population by chance has all fixed characters set to ‘1’, for which the probability is about one in a thousand (1/2^10^ with learning) rather than about one in a million (1/2^20^ without learning), selection can optimize the string via the usual process of gradient ascent in a smooth fitness landscape.

According to this model, learning satisfies the formal conditions of Montévil & Mossio [35] for being a constraint on the slower process of selection. First, because it catalyses evolution and thus has an asymmetrical effect on it. Second, because although selection affects the potential to find the target by determining which characters are fixed or modifiable, the learning process itself (randomly flipping the modifiable states) is unaffected. According to these criteria the (asymmetrical and symmetrical) conditions must be met at the same timescale, as they are here because the search for the target is accelerated by learning at the (inter-generational) timescale at which selection occurs, and because the mechanism of learning persists also at that timescale.

Defined in this way, constraints can be both limiting (they ‘do not generate new possibilities for the constrained process’ [35, p. 183]) and generative (they ‘enable outcomes that would otherwise be improbable’ [35, p. 183]), and this apparent contradiction is clearly resolved by Hinton & Nowlan’s model. Consider that while learning makes the all but impossible search for the target quite possible, it does not generate new possibilities for selection—if the first character of all in the initial population was fixed at ‘0’ then no amount of recombination could be successful. As such, learning in this model constitutes a generative constraint on selection. Selection is in turn a constraint on learning, given that the success of the evolved system rests in its ability to allow the target to be quickly learnt.

Having identified a computational model that represents mutually constraining slow and fast processes, we will next show how these ideas can be used to represent constraint closure in a more general computational model, which permits the basic effect established by Hinton & Nowlan to be applied to more elaborate systems of mutually constraining processes.

### (b) A computational model of constraint closure

Consider a system comprising two interacting processes, one slow (*A*) and one fast (*B*). The configuration (or ‘state’) of the system is given by a string of *N* binary variables, and the designation of each to either timescale. At a given step at the slower timescale, each variable is either in state 1 or state 0, or it is variable on the faster timescale, which we denote with the ? symbol.

The target state for the system is for all variables to be in state 1. (Note that the specific configuration of the target state is irrelevant, but choosing the target to be all 1 makes the model easier to describe and simulate, and simpler to analyse.) The slow process, *A*, reconfigures the system at each iteration of the slower timescale, by randomly allocating variables to either timescale with probability *p*_*A*_, and accepting those changes with probability *q*_*A*_.

For each iteration of the slow process, a clock is reset to *t*_*B*_ = 0 and then incremented through *T*_*B*_ steps, defining the faster timescale. On each increment of *t*_*B*_, the binary state of all ? variables is set at random, the clock is stopped if the system acquires the target state, and the acquisition rate is defined as *τ*_*B*_ = *t*_*B*_/*T*_*B*_, else *τ*_*B*_ = 1. (Note that *τ*_*B*_ will always be 1 if any variables are 0.) The configuration trialled by the slow process is then accepted with probability *q*_*A*_ = *σ*[Δ*τ*_*B*_(*t*_*A*_)], where *σ*[*x*] = (1 + e^*kx*^)^−1^, Δ*τ*_*B*_(*t*_*A*_) = *τ*_*B*_(*t*_*A*_) − *τ*_*B*_(*t*_*A*_ − 1), and *k* is a (high) temperature parameter. Accordingly, if the configuration increases the time taken to acquire the target state (on the faster timescale) that configuration is very likely to be rejected (on the slower timescale). Setting $p_{A}=\frac{1}{2}\tau_{B}$ ensures that the system is reconfigured by the slow process on an increasingly local scale as the target becomes more rapidly acquired.

The slow process *A* constrains the fast process *B* insofar as the number of characters assigned to timescale *A* determines the size of the search space for *B*, and thus the time it will probably take to acquire the target. The fast process in turn constrains the slow process because the change in the rate of acquisition, measured between successive iterations on timescale *A*, determines the direction (*p*_*A*_) and magnitude (*q*_*A*_) of changes trialled by process *A* (and evaluated through the dynamics of *B*).

### (c) Finding the needle in the haystack by constraint closure

**Figure 1.**
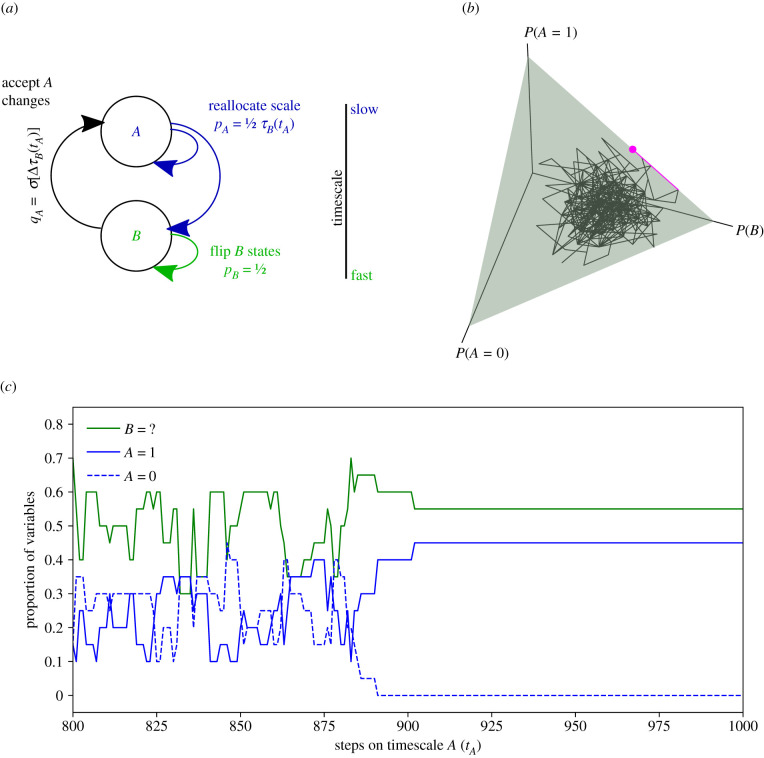
Closure of constraints in a system of two dynamical processes. (*a*) Model architecture for a system composed of a slow dynamical process (*A*) and a fast dynamical process (*B*), showing the probabilities of modifying states at each timescale (*p*_*A*_ and *p*_*B*_), and the probability of those modifications being accepted (*q*_*A*_). (*b*) The state space can be visualized as a triangular plane, shown in green (*P*(1) + *P*(0) + *P*(?) = 1), wherein the system initially drifts at random (black trace) before discovering the edge *P*(1) + *P*(?) = 1 along which *τ*_*B*_ < 1 is graded and thus the evolution of the system becomes guided (pink trace terminating in a pink dot). (*c*) Time evolution of the system, corresponding to the trace in (*b*), showing that once the target state is first acquired (at *t*_*A*_ = 884) variables quickly become reallocated from the fast process to the slow process, speeding subsequent acquisition (in a manner comparable with that described by [42]). For this simulation *N* = 20, *k* = 50, *T*_*A*_ = *T*_*B*_ = 1000.

When the dynamics of this model are simulated (figure 1), the system quickly settles on a configuration in which all of the specified variables reach a state of 1, with the unspecified variables allowing the target configuration (all 1s) to be acquired in a relatively small number of iterations of the fast dynamics.

We can understand this effect by considering the state space in which the dynamics operate (figure 1*b*). Consider that the proportion of the *N* variables that are in state 1 at timescale *A*, denoted *P*(1), together with proportions at state 0, *P*(0), and unspecified, *P*(?), must combine to equal 1. The allocation of variables to the faster timescale thus extends the space of possible configurations of the system from the line *P*(1) + *P*(0) = 1 to the plane *P*(1) + *P*(0) + *P*(?) = 1. The boundary of the plane has the shape of an equilateral triangle (figure 1*b*), with its vertices at coordinates (1.0, 0.0, 0.0), (0.0, 1.0, 0.0) and (0.0, 0.0, 1.0), and with the target state at the vertex where *P*(0) = *P*(?) = 0.

From the perspective of the slow process, viable solutions are only to be found along the edge of this triangle where *P*(0) = 0. The behaviour of the system is initially dominated by the dynamics of this slower process as it drifts about on this plane, failing to acquire the target configuration. However, as *τ*_*B*_ = 1 and thus the probability that variables are reallocated between *A* and *B* is maximum ($p_{A}=\frac{1}{2}$), the system samples the space by taking large (undirected) strides, and the chance of it finding the edge where *P*(0) = 0 is fairly high, i.e. by replacing all 0 variables with 1 variables at timescale *A* or reallocating them to timescale *B* as ?s. Once at this edge, the system has the opportunity to acquire the target state via the dynamics of the fast process, such that *τ*_*B*_ < 1, and the modification is thus likely to be accepted by the slow process (*q*_*A*_ > 0.5).

Finding this edge creates an *explicit* pressure on the system to reduce the proportion of undecided variables, i.e. a pressure in the −*P*(?) direction, because *τ*_*B*_ is proportional to the probability of acquiring the target state 1/2^*P*(?)^. This explicit pressure in the −*P*(?) direction, in combination with the constraint that viable configurations are bound to the edge *P*(0) = 0, which is oriented at 45 degrees to it, thus creates an *implicit* pressure in the +*P*(1) direction, causing the system to hug this boundary as it evolves towards converting timescale *B* variables to timescale *A* variables in state 1. This part of the system’s trajectory is highlighted in figure 1*b* in pink. The result is that the system reaches the target configuration quickly. The search for a single state (all 1) in a space of 2^*N*^ configurations becomes straightforward.

This system behaves in essentially the same way as the population model of Hinton & Nowlan, and as reported in their original paper we can see in figure 1*c* that when the target state is first acquired the variables allocated to timescale *A* and in state 1 quickly start to replace those in state 0, as well as some of those allocated to timescale *B*. Indeed the evolution of the Hinton & Nowlan model can be visualized in exactly the same way as we have shown in figure 1*b*, by plotting the proportion of each character in the population in the same triangular plane. The main differences are that in their model (i) the slow (evolutionary) process is realized by a population rather than a single string (see [46,47] for similar proposals), and (ii) the increasingly localized search of the space that occurs as the system approaches the solution is achieved via recombination.

**Figure 2.**
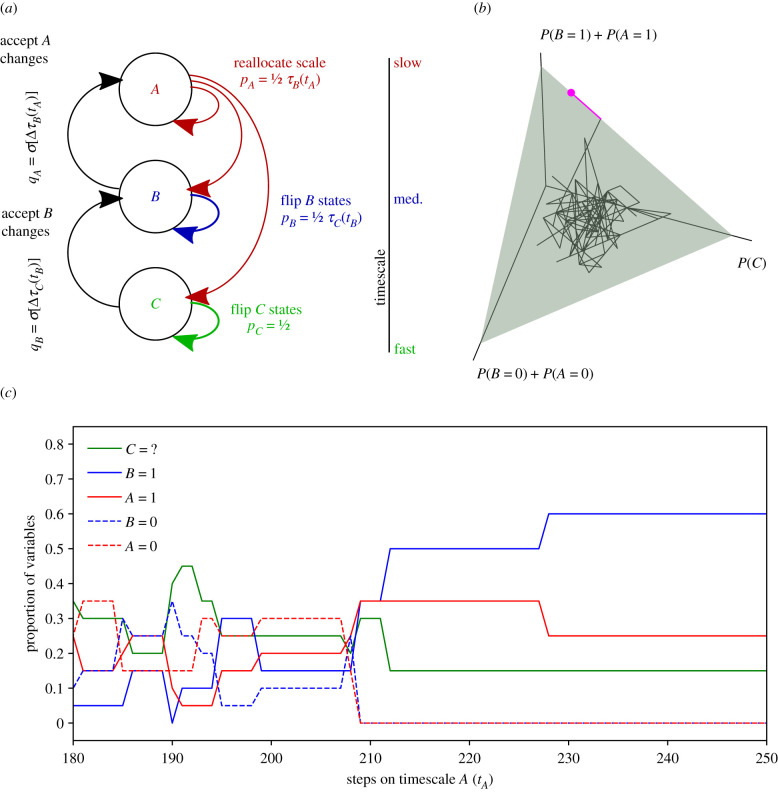
Closure of constraints in a system of three dynamical processes. (*a*) Model architecture for a system composed of a slow (*A*), medium (*B*) and fast (*C*) dynamical process. (*b*) The state space can be visualized as a triangular plane, shown in green (*P*(1) + *P*(0) + *P*(?) = 1, where *P*(0) and *P*(1) are the proportion of system variables in state 0 or 1 that are allocated to either process *A* or *B*, and *P*(?) is the proportion allocated to process *C*). (*c*) Time evolution of the system, corresponding to the trace in (*b*), showing that once the target state is first acquired (at *t*_*A*_ = 209), variables quickly become reallocated from the fast process to the slow process, before being further reallocated from *A* to *B*). For this simulation *N* = 20, *k* = 50, *T*_*A*_ = 1000 and *T*_*B*_ = *T*_*C*_ = 5000.

This generalization of the essential dynamics of Hinton & Nowlan’s model permits the study of more elaborate configurations of reciprocally constraining processes, including an extension to three processes that is depicted in figure 2 and elaborated in the electronic supplementary material, appendix S1.

## 4. Faster processes can scaffold slower processes

The key aspect of constraint closure that has been addressed here is how faster processes can usefully constrain slower processes. Interestingly, the modelling reveals how constraint closure can allow for specific configurations spread over multiple timescales to become consolidated in the dynamically maintained configuration of the states of slower processes. We suggest the concept of a ‘scaffold’ as a useful metaphor for how this consolidation takes place.

In real terms, a scaffold is a quickly built and temporary structure that facilitates the more gradual construction of a more permanent structure. In the short-term, the scaffold solves a similar problem as does the object whose construction it enables, for example to elevate workers, but the properties that allow it to be useful in many different contexts are also the properties that allow it to solve any particular problem only partially and only temporarily.

This description applies equally well to the interaction between timescales in Hinton and Nowlan’s model of the Baldwin effect and to the generalization developed here. Random bit-flipping as a representation of learning that takes place within each lifetime enables fitness peaks to be reached more easily, by dividing the search space between two (ontogenetic and phylogenetic) timescales. While the solution is transient and lost with the death of the individual that discovers it, the height from which that individual began its ascent is retained as an increment in the organization that it enables to be constructed more slowly by natural selection. Dennett used the crane (in contrast to a sky-hook) as a metaphor for the effect represented by Hinton & Nowlan’s model [45], and while cranes and scaffolds are compatible metaphors, scaffolding additionally connotes the important sense in which the facilitating structure must itself also be dynamically constructed. Bickhard [48] proposes a similar use of the term scaffold to describe processes than enable intermediate points of construction in evolution and development.

Let us define the concept of a scaffold more carefully, and in the broader context of constraint closure that is suggested by the modelling results. We propose that of two processes, that with the faster dynamics, *B*, can serve as a scaffold for the organization of the slower process, *A*, if the functional consequences (or mere existence) of process *B* constrain the dynamics of process *A*, such that some product of process *A* may be improved (or merely permitted) by modification to the organization of process *B*. Thus, in the current model (figure 1), process *B* serves as a scaffold for process *A*.

Note that according to this definition, scaffolding is a possible consequence of constraint closure, and one that is to be expected in the specific case where the product of a slower process may be associated *a priori* with some utility, i.e. some criteria against which its usefulness to the maintenance and/or persistence of the system may be evaluated (and thus improved). But note also that, as in Hinton & Nowlan’s model, a scaffold can be useful (by partitioning a search space) before any measure of that utility becomes available to the system.

Next we explore scaffolding in the layered architecture of the mammalian brain with respect to two exemplar processes—first, physiological regulation, and the specific example of social thermoregulation, through which we refine the concept of scaffolding further still, and second, visual attention.

## 5. Constraint closure in physiological regulation

In thermoregulation for cold defence, regulatory systems such as skin vasoconstriction, thermogenesis (fat burning) and shivering, operate via independent thermoeffector loops, with distinct sensing and effector branches, and with different thermal sensitivities related to their metabolic costs [33,49–51]. These mechanisms, which employ a mixture of feedback, feed-forward and open loop control [32,33,52,53], are hypothesized to have evolved one-at-a-time [49,50], to provide added layers of robust control [54], without reference to a common measure of body temperature [33].

Many of the key determinants of successful thermoregulation involve actions that influence the ambient temperature, for instance, by moving to an environment that is less thermally stressful, or by engineering the local environment. These actions can vary from those as simple as moving along a temperature gradient, to more complex behaviours such as nest-building, wearing clothing, and seasonal migration. Social behaviours, such as attachment [55], social touch [56] and huddling with conspecifics [57], are also critical to effective temperature maintenance in mammals, particularly during infancy when adult regulatory mechanisms may be only partially developed [58]. Whereas brainstem and subcortical systems (including the hypothalamus) largely drive instrinsic mechanisms for thermoregulation [33,50], forebrain systems for sensory discrimination, emotion, learning and planning, play a critical role in thermoregulatory behaviour [59]. Indeed, the insular and cingulate cortices have been described as forming a ‘homeostatic sensorimotor cortex’ [60] that sits at the top of the layered brain architecture for physiological regulation. This interplay of subcortical and cortical systems shapes, and is in turn shaped by, thermal cues associated with social interactions experienced in infancy [61].

### (a) Social thermoregulation

An informative example of social thermoregulation is provided by huddling in infant mammals such as in mouse or rat litters [57,62–68]. The huddle is a dynamical process, which results from the (local) heat-seeking or heat-avoidance of individuals, from which adaptive group-level (global) dynamical properties emerge at a slower timescale. These include a widening of the thermoneutral zone, i.e. of the range of ambient temperatures over which core temperatures can be maintained at minimal metabolic cost [51,69], with the width of the thermoneutral zone corresponding with the steepness of a sigmoidal relationship between ambient temperature and huddle size that has been measured in experiments with mice [68]. This relationship is evidence that huddle formation constitutes a second-order phase transition, which occurs at an ambient temperature 10–20°C lower than the core body temperature defended by the individuals that comprise the huddle. Successful recreations of these properties in agent-based models, which describe the thermal dynamics of the huddle as an emergent property of self-organizing interactions between the individuals, underscore how dynamical systems can maintain homeostasis without maintaining explicit representations of set-points [70,71].

Viewed in terms of ‘aggregons’—the distribution of *n* individuals amongst physically distinct contact groups [66,67]—huddling is a trajectory through a state space in which the possibilities are given by the integer partition of *n*. As aggregons can be grouped by thermal macrostate (grouping *n* = 6 into a 2 and a 4 is thermally equivalent to grouping them into a 4 and 2), a similar analysis to that developed above, based on a more general partition of binary variables, can also be applied to huddling dynamics. Accordingly, the probability with which aggregon macrostates are reconfigured on the slow timescale (corresponding to probability *q*_*A*_ in the model developed in §2) is determined by the thermal consequences of the individual behaviours that modify microstates on the fast timescale. Under this assumption, simulated huddling also recreates the experimentally observed huddling sigmoid, as well as a widening of the thermoneutral zone [71,72]. Thus, fast thermotaxic orienting behaviours of the individuals scaffold slow group-level thermoregulatory dynamics.

Huddling can in turn scaffold thermoregulatory dynamics on at least two slower timescales. First, as the capacity for physiological thermoregulation matures over the first postnatal month, the preferred ambient temperature of infant mice reduces from around 38°C to around 32°C [73], transitioning from an ectothermic physiology, like that of reptiles, to the endothermic profile of the adult. Huddling is at first driven by thermal cues, but later is instead driven by social cues. Fillial huddling behaviours, i.e. maintaining contact with an object of neutral temperature, can be induced in older animals by an unusual odour experienced, during an early critical period, while in contact with an object that was warm (and soft) [74–78]. Thus physiological huddling, driven by thermal cues and an immature thermal physiology in neonates, provides a scaffold for the development of fillial huddling preferences, via the relatively slow process of odour-heat conditioning [72]. On a similar ontogenetic timescale, individual differences in early huddling behaviours have also been shown to be correlated with differences in various other measures of adult sociality (and locomotor development) [79–81].

**Figure 3.**
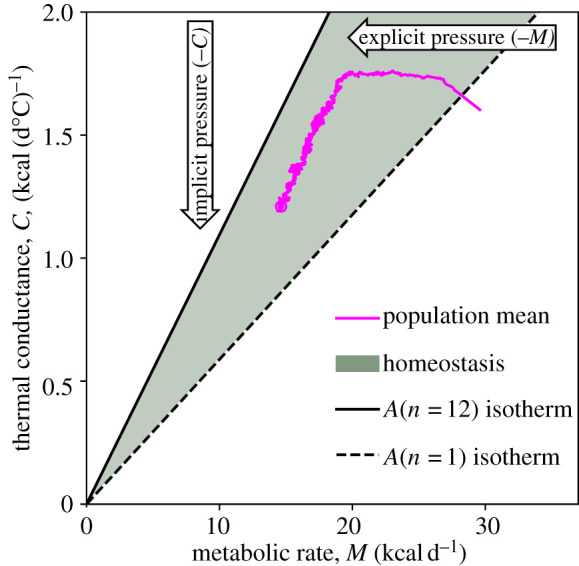
Scaffolding through social thermoregulation. Simulated evolution of the thermal physiology in a population of 100 mouse litters, each comprising *n* = 12 littermates, using the model described by Glancy *et al*. [82]. Genetic variables describe the basal metabolic rate *M* and thermal conductance *C*. The population mean is shown evolving over time (generations) in pink. The green region shows the range of thermal physiologies over which thermal homeostasis can be maintained at minimal metabolic cost. This region is bounded by isotherms determined by the exposed body surface area, *A*(*n*) = *n*^−1/4^, of mice that are maximally huddled, *A*(*n* = 12), or fully exposed, *A*(*n* = 1). When the population is first able to thermoregulate, an explicit selection pressure in the −*M* direction pushes it to the huddling isotherm. Once there, drifts in the thermal physiology of the population are inconsequential only towards −*C*, creating an implicit pressure in this direction, where the thermal physiology increases the *opportunity* for mutations to further reduce metabolic cost in future generations.

Second, huddling can scaffold thermoregulatory dynamics on a phylogenetic timescale (figure 3). To see this, we use Newton’s Law of cooling to write *M* = *AC*(*T*_*b*_ − *T*_*a*_), with *M* the metabolic rate, *A* the proportion of the body that is exposed, *C* the thermal conductance, *T*_*b*_ the body temperature, and *T*_*a*_ the ambient temperature [69]. In a ‘genome space’ with dimensions for *M* and *C*, thermal homeostasis (*T*_*b*_ = 37°C) occurs along the isotherm *M* = *AC*(37 − *T*_*a*_). In the extreme, mutations that modify the metabolism or thermal conductance in either direction would fail if they caused the physiology to deviate from the isotherm, to where an animal would fail to thermoregulate. However, huddling allows the exposed surface area of each animal in a litter of size *n* to be reduced to approximately *A*_min_ = *n*^−1/4^ [68], which creates a new isotherm, *M* = *C*(37 − *T*_*a*_)*n*^−1/4^, and a region between the two in which thermal homeostasis can be achieved for a wide range of thermal physiologies. Within these bounds, selection pressure to reduce the metabolic costs of endothermy is an *explicit* pressure in the −*M* direction, with evolution pushing the thermal physiologies of huddling species towards the new isotherm.

The crucial idea that allows huddling to scaffold the evolution of thermal physiology, is that this new boundary is *oriented* in the state space relative to the direction of the explicit selection pressure (−*M*), creating a new *implicit* selection pressure towards −*C* [82]. To see this, consider that for a littermate otherwise poised on the huddling isotherm, a mutation that affects the metabolic rate, *M*, causes either death by cold or a reduced metabolic efficiency that will be selected out. A mutation that increases thermal conductance, *C*, such as thinning the coat, also causes death by cold, but a mutation that reduces *C* is inconsequential, because adjustments in thermal behaviour can compensate. As the mutation of *C* incurs no additional metabolic cost, the *explicit* selection pressure on *M* is unchanged, and the mutation survives. But as the orientation of the huddling/death-by-cold isotherm is oriented, this drift towards −*C* creates a new *opportunity* for subsequent mutations to further reduce *M*, which can be measured in terms of the gradient of the isotherm, i.e. d*M*/d*C* = (37 − *T*_*a*_)*n*^−1/4^. As such, social thermoregulation creates a scaffold for the evolution of thermal physiology, via the relaxation of metabolic constraint, and the opportunity afforded by this scaffold increases with the litter size.

In more general terms, we can refine the idea of a scaffold as follows; scaffolding requires a correlation between the variables of a system that bound its dynamics, such that otherwise random variation in one variable becomes directed by the opportunity it affords for work to be done on another.

### (b) The layered control architecture for human visual attention

That the sensory, motor and motivational capacities of the neonate are largely supported by subcortical systems, which scaffold the development of cortical systems, is well illustrated by the example of human visual attention.

At birth the control of eye movements is dominated by the midbrain superior colliculus (SC), which, together with other brainstem circuits, can identify targets for fixation, generate saccadic eye movements, and track a moving object through a sequence of saccadic shifts [24,83–85]. This system, which is low-definition and favours the visual periphery [84], is homologous to the optic tectum (OT) of vertebrates such as reptiles and amphibians, that lack a well-developed cortical visual system [86].

During infancy, the capacity to generate complex eye movements, including saccades to remembered stimuli and to perform smooth pursuit of moving targets, develops over the first three to four months as part of a gradual shift in the control of visual attention to the cortex [12,83,84,87]. This orienting capability emerges alongside better discrimination of visual features and improving classification of stimulus objects by the ventral visual stream [12]. Beginning with activity in the deep layers of primary visual cortex (V1), cortical control of visual attention gradually expands to include more processing layers, and additional cortical regions that project to the SC, including the middle temporal area and the frontal eye fields [12,84]. Control of visual orienting by these developing structures primarily operates by modifying the selection of targets in the downstream midbrain SC [5,84,88]. Unexpected loss of cortical vision leaves the subcortical system largely intact and capable of supporting some visual guidance of behaviour without conscious awareness (termed blindsight) [89]. These observations collectively demonstrate that the cortical/sub-cortical partitioning of brain mechanisms for visual attention meets the definition of a layered control architecture.

The early-developing collicular orienting system has some significant, though poorly-studied, ascending connections [90]. However, this system also appears to scaffold the development of cortical vision less directly by selecting targets for attention, and by controlling eye movements and fixations. In this way, the neonate SC helps to determine what the cortical system will see and thus what it will learn. This structuring of the information available to the developing cortex is critical to enabling normal development [91,92]. For example, an important set of targets for infant fixations are the faces of conspecifics, and particularly of the child’s carers. Newborn humans show immediate interest in face-like stimuli, which includes preferential looking towards human faces, animal faces and graphic designs or toys that include face-like features [93]. Each of these stimuli appear to trigger target selection by the collicular orienting system which has been described as having a template for faces that could be as simple as ‘three high-contrast blobs in the correct locations for two eyes and a mouth’ [93, p. 170]. By contrast, the capacity to discriminate between the faces of conspecifics, and thereby to recognize individual faces, emerges from around two months and is dependent on the separate cortical visual learning system [93].

**Figure 4.**
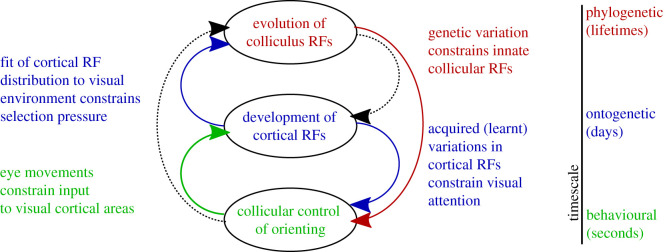
Scaffolding a layered control architecture. In the example of visual attention, there are three relevant timescales to consider. The fastest timescale corresponds with behaviour, where an epoch of the relevant dynamics might be measured in terms of the interval between visual saccades. The intermediate timescale corresponds with postnatal development, where an epoch might be measured in terms of days or weeks, as learning shapes neuronal receptive fields (RFs). The slowest timescale corresponds with evolution, where epochs might be measured in terms of lifetimes, between which innate functionality and patterns of inter-area connectivity adapt. Dotted lines represent constraints that exist but are not necessarily required for constraint closure in this system.

Viewed as a constraint cascade we might consider the subcortical attention system that involves the SC to be a ‘slow’ system as its function is, for the most part, innately specified and therefore adapts over evolutionary time-scales. However, the same system can also be considered as a fast process since patterns of SC activity direct orienting on the millisecond timescale of visual saccades. By determining attentional targets in infancy the SC constrains the development of the more slowly developing cortical system by structuring its sensory experience [91]. Cortex, in turn, constrains the very slow dynamics of subcortical evolution. Specifically, by gradually imposing its own targets for visual attention—determined by feature analysis, classification, sensor fusion and memory—cortex constrains the future evolution of the subcortical orienting system, relaxing requirements for innate systems that support these, now acquired, capacities. Indeed, in the evolution of SC there is evidence of encephalization of some of the functions of ancestral OT [94]. We can therefore think of constraint closure in the evolution and development of orienting systems as operating across three distinct time-scales as illustrated in figure 4.

## 6. Discussion

From the perspective of the theoretical paradigm of constraint closure, an important challenge is the requirement for processes with faster dynamics to be constraints on processes with slower dynamics (see §2 and [35]). It is intuitive to think of systems in which slowly changing (or fixed) variables influence more rapidly adapting variables, for example, as the boundary shape of a cell or tissue constrains the patterns of reaction and diffusion that form within it [95], or as the pattern of connections in a neuronal network constrains the pattern of action potentials that it generates. However, it is perhaps less intuitive to think of slower systems as being constrained by faster ones. In this article we have explored and developed the idea of constraint closure in biological systems, drawing on the Hinton & Nowlan model to show how processes at different time-scales can constrain each other, and in particular how fast processes are able to usefully constrain slower processes, without requiring the state acquired by the faster process to be communicated directly to the slower process. We hope this work will help further the theoretical development of the notion of constraint closure.

### (a) Time-scales of constraint closure in brain evolutionary-developmental biology

Our investigation also demonstrates the relevance of constraint closure to the field of evolutionary-developmental biology (evo-devo) [96,97], that investigates how processes across multiple ontogenetic and phylogenetic timescales constrain each other to generate robust and adaptive designs for organisms, and contributes to the growing body of computational modelling in this domain (see [98] for review). In terms of applying this framework to our understanding of the brain, our findings also show the need to go beyond thinking of the brain in terms of neural structures and circuits; rather we must also think in terms of the various timescales over which these subsystems are dynamically reorganizing. For instance, we noted that two distinct processes are attributable to the subcortical circuitry for visual orienting involving the SC—a fast process by which it directs attention and thus constrains cortical development, and a slow process by which its functional organization evolves. In the future, it would be interesting to explore how this framework could be applied to understanding time-scales of learning in multilayered neural networks such as recurrent network models of cortical systems (e.g. [99]).

### (b) Scaffolding and relaxed selection

In simplifying and generalizing the Hinton & Nowlan model, we have also clarified, in geometrical terms, how learning partitions organismal design space [100] and creates gradients for evolutionary and developmental processes to climb. We have suggested scaffolding as a useful metaphor for this effect, and as a more general property of systems with constraint closure. This idea also serves to tie together physiological and cognitive mechanisms, for example, as described in §5b with regard to the emergence of filial huddling from physiological regulation via scaffolding. As such, it could help progress ongoing debates about the grounding of cognition in metabolism (e.g. [101]).

Scaffolding can operate in multiple ways, one of which is the Baldwin effect whereby fast learning processes scaffold slow evolutionary processes. However, other forms of scaffolding, for instance via behavioural support from conspecifics [48], or from restructuring of the physical or sociocultural environment, [102,103], may also *relax* selection [104] for rapid learning, allowing processes that might otherwise be specified innately to be susceptible to learning and therefore able to better tune themselves in context. Scaffolding, in this form, can lead to a ‘reverse Baldwin effect’ [46] which appears to have driven increases in brain size in mammals, primates, and humans; with encephalization of function in these species trading prolonged immaturity, and a requirement for dedicated familial support, for the increased flexibility of an enlarged cortex that can better adapt to the world in which it finds itself [105].

### (c) Hierarchical and layered control systems

There is a long history of understanding brain organization and the control of behaviour from the perspective of hierarchy. In a hierarchy of *control* there is strict flow of command from top-to-bottom, characterized by the relationship that each higher unit is the ‘boss of’ one or more lower units [106]. An alternative meaning of hierarchy relates to hierarchies of embedment, containment, or classification [106,107], in which a system is considered as composed of parts, which are themselves composed of smaller parts, and so on. Hierarchies of embedment have multiple benefits including the capacity to partition a search space to facilitate faster discovery of solutions [107,108] in a manner similar to that explored here.

The view of constraint closure explored in this article implies that the relationships between sub-systems in the brain, including those between cortical and sub-cortical systems, are not adequately captured by a strict notion of a hierarchy of control because subsystems are mutually constraining at different time-scales. The concept of layered control, on the other hand, allows for a configuration of sub-systems in which constraints can operate between levels, in both directions, and across multiple timescales (see also [4,40]), while also benefiting from modular decomposition of control and robustness through resistance to damage (dissociations) [4,16]. It is worth noting that in the neuroscience literature the term hierarchy is sometimes used more loosely to describe an organization that is not a strict control hierarchy, often not distinguishing between hierarchies of embedment and command. However, since we are concerned here with the brain’s control architecture we consider that this distinction between hierarchical and layered control is worth emphasizing.

In a similar way, some models of physiological regulation have proposed a form of a hierarchy, with predictive allostatic systems acting to modify the set points of feedback-controlled homeostatic systems (e.g. [109], see [110] for review). However, the brain does not appear to explicitly represent physiological set points in the way envisaged [29,32,33]. In the place of such a hierarchy, regulation might be better imagined as a closure of constraints between multiple processes with dynamics that operate on different timescales. The modelling example developed here is useful from this perspective, in demonstrating how a target state for the overall configuration of a system need not be represented explicitly at any processing layer. In other words, physiological ‘balance points’ may be usefully seen as the implicit outcomes of the parallel operations of multiple predictive, feedback and open loop sub-systems in the brain and body, that are extended through behavioural-environmental loops. The literature on dynamic systems and situated robotics provides multiple examples of the emergent regulation of an uncontrolled variable, in a distributed system, in a manner that supports robust control as envisaged here [16,111,112]. With respect to physiological regulation specifically, Bich *et al.* [34,40,113] have similarly proposed that for such a system to regulate itself requires a dynamically decoupled sub-system of constraints that responds to external perturbations by instantiating a new dynamical regime that is better able to maintain stability in the presence of those perturbations.

## 7. Conclusion

This view of neural processes, as components of multi-layered temporally extended systems for controlling bodily and behavioural functions, sees brain organization as an emergent property of dynamically maintained constraints between sub-systems that are reciprocally generative for, and dependent on, one another. In placing some of these constraints in the body, the environment and the social milieu, it conforms with embodied and extended views of cognition while also helping to understand how systems of such complexity as the human brain can emerge through evolution and development.

## Data accessibility

The model code is included as the electronic supplementary material.
